# Acute onset supraclavicular lymphadenopathy coinciding with intramuscular mRNA vaccination against COVID-19 may be related to vaccine injection technique, Spain, January and February 2021

**DOI:** 10.2807/1560-7917.ES.2021.26.10.2100193

**Published:** 2021-03-11

**Authors:** María Fernández-Prada, Irene Rivero-Calle, Ana Calvache-González, Federico Martinón-Torres

**Affiliations:** 1 Vaccines Unit, Preventive Medicine and Public Health Department, Vital Álvarez-Buylla Hospital, Health Care Service of Asturias, Mieres, Spain; 2 WHO Collaborating Centre for Vaccine Safety, Santiago de Compostela, Spain; 3 Translational Pediatrics and Infectious Diseases, Hospital Clínico Universitario and Universidad de Santiago de Compostela (USC), Santiago de Compostela, Spain; 4 Genetics, Vaccines and Pediatric Infectious Diseases Research Group (GENVIP), Instituto de Investigación Sanitaria de Santiago and Universidad de Santiago de Compostela (USC), Santiago de Compostela, Spain; 5 Digestive Surgery Service, Hospital Clínico Universitario de Santiago de Compostela, Santiago de Compostela, Spain

**Keywords:** COVID-19, mRNA vaccine, Vaccine, adverse events, supraclavicular lymphadenopathy

## Abstract

Monitoring adverse reactions following immunisation is essential, particularly for new vaccines such as those against COVID-19. We describe 20 cases of acute onset of a single supraclavicular lymphadenopathy manifesting between 24 h and 9 days after ipsilateral intramuscular administration of an mRNA-based COVID-19 vaccine, referred to our WHO Collaborating Centre for Vaccine Safety. Our results indicate that the swelling of supraclavicular lymph nodes following immunisation may constitute a benign and self-limited condition, related to a higher than recommended injection site.

The monitoring of adverse reactions associated with vaccination is one of the most important factors in vaccine safety. Although vaccines are among the safest drugs currently on the market, vaccines are not completely risk-free, and adverse events may occur following vaccination. Careful assessment of any adverse events following immunisation is essential to distinguish those that are causally linked to the vaccination from those just coincident in time, in order to prevent vaccine distrust or misperceptions [1]. The objective of this rapid communication was to report a series of adverse reactions consisting of acute onset supraclavicular lymphadenopathy coinciding with vaccination against coronavirus disease (COVID-19).

## Case series 

Here we describe a series of 20 clinical cases referred to our World Health Organization (WHO) Collaborating Centre for Vaccine Safety, reporting the acute onset of a single supraclavicular lymphadenopathy coinciding with the ipsilateral intramuscular administration of a dose of an mRNA vaccine (Figure). All patients were female healthcare workers and had received between 15 January and 22 February 2021, an ipsilateral intramuscular administration of an mRNA-based vaccine against COVID-19 (Comirnaty, Pfizer–BioNTech (Puurs, Belgium) in 19 cases or mRNA-1273, Moderna (Madrid, Spain) in one case), with different administration days and vaccine lot numbers. 

**Figure:**
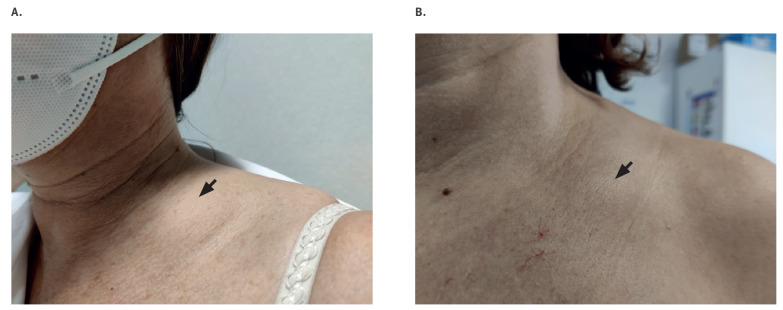
Supraclavicular lymphadenopathy ipsilateral to the vaccination arm (left), COVID-19 vaccination, Spain, 2021

A. The lymphadenopathy appeared 1 day after the first vaccine dose, and it is in resolution, with significant reduction in size since onset.

B. The lymphadenopathy appeared 7 days after the second vaccine dose and it is in resolution.

All 20 cases were women, with ages ranging from 25 to 60 (median: 44) years (Table). None had a history of severe or unusual adverse reactions following immunisation. Two of the cases had a prior history of thyroid disease (follicular adenoma and thyroid cancer), a third case was diagnosed with autoimmune thyroiditis in the course of the diagnostic work-up, and another two cases had Sjögren syndrome (one associated with hypertension and the other with systemic lupus erythematosus). In five patients, fine needle puncture aspiration was performed with results showing reactive inflammatory signs, with lymphocytic infiltrate and active germinal centres. In one patient, a mammography was also performed, with no pathological finding. All lymphadenopathies had inflammatory symptoms (pain, swelling), were rounded and mobile, and all but one appeared in the first 24 h to 9 days after vaccine administration. The remaining patient detected the adenopathy 24 days after vaccination, prompted by contact with another affected case, but she retrospectively recognised minor local symptoms in the preceding days. 

**Table:** Characteristics of healthcare workers with supraclavicular lymphadenopathy after COVID-19 vaccination, Spain, 2021 (n = 20, all female)

Number	Age group (years)	Clinical history		COVID-19 history (date of diagnosis)	ARNm vaccine name (vaccine lot number)	Dose	Side of injection	Onset date of symptoms (days since vaccination)	Duration of symptoms (outcome)	Medical examinations
1	30–35	No		No	Pfizer–BioNTech (EJ6134)	2nd	Left	28 Jan 21 (1 day)	5 days (resolved)	None
2	30–35	No		No	Pfizer–BioNTech (EJ6134)	2nd	Left	30 Jan 21 (1 day)	10 days (resolved)	None
3	25–30	No		No	Pfizer–BioNTech (EK9788)	1st	Left	21 Jan 21 (2 days)	7 days (resolved)	Yes ^a^
4	30–35	No		Yes (5 Nov 2020)	Pfizer–BioNTech (EK9788)	1st	Left	1 Feb 21 (1 day)	6 days (resolved)	None
5	45–50	No		No	Pfizer–BioNTech (EL8723)	2nd	Left	4 Feb 21 (1 day)	9 days (in resolution)	None
6	45–50	No		No	Pfizer–BioNTech (EK9788)	2nd	Left	31 Jan 21 (0 days)	15 days (in resolution)	None
7	25–30	No		No	Pfizer–BioNTech (EL8723)	2nd	Left	8 Feb 21 (3 days)	5 days (resolved)	None
8	55–60	No		No	Pfizer–BioNTech (EK9788)	2nd	Left	5 Feb 21 (1 day)	(15 days) (resolved)	None
9	55–60	No		No	Pfizer–BioNTech (EK9788)	2nd	Left	9 Feb 21 (7 days)	30 days (in resolution)	None
10	55–60	Yes	Sjögren syndrome, arterial hypertension	No	Pfizer–BioNTech (EK9788)	1st	Left	10 Feb 21 (9 days)	10 days (resolved)	None
11	35–40	Yes	Thyroid follicular adenoma	No	Pfizer–BioNTech (EM0477)	2nd	Left	5 Feb 21 (3 days)	9 days (resolved)	Yes ^a^
12	40–45	No		Yes (20 Jan 2021)	Pfizer–BioNTech (EK9788)	2nd	Left	10 Feb 21 (1 day)	8 days (resolved)	None
13	45–50	Yes	Autoimmune thyroiditis	No	Pfizer–BioNTech (EM0477)	1st	Left	21 Jan 21 (6 days)	24 days (in resolution)	Yes ^a^
14	50–55	No		No	Pfizer–BioNTech (EK9788)	1st	Left	30 Jan 2021 (1 day)	7 days (resolved)	None
15	35–40	No		No	Pfizer–BioNTech (EK9788)	2nd	Left	22 Jan 2021 (1 day)	16 days (resolved)	Yes^b^
16	55–60	Yes	Systemic lupus erythematosus, Sjögren syndrome	No	Pfizer–BioNTech (EL8723)	2nd	Left	7 Feb 2021 (2 days)	10 days (resolved)	None
17	50–55	No		No	Pfizer–BioNTech (EM6950)	2nd	Left	18 Feb 2021 (1 day)	5 days (resolved)	None
18	45–50	Yes	Asthma and thyroid cancer	No	Pfizer–BioNTech (EP9598)	2nd	Left	24 Feb 2021 (2 days)	7 days (in resolution)	None
19	35–40	No		No	Pfizer–BioNTech (EK9788)	2nd	Left	26 Feb 2021 (24 days)	30 days (in resolution)	None
20	35–40	No		No	Moderna (300042460)	1st	Right	22 Jan 2021 (4 days)	32 days (in resolution)	Yes^a^

^a^ Ultrasonography and fine-needle aspiration puncture.

^b^ Ultrasonography, mammography, and fine-needle aspiration puncture.

In six cases, the symptoms occurred after the first vaccine dose, while the remaining 14 happened after the second dose. All of them completed the two-dose vaccination schedule. Twelve of the 20 patients spontaneously reported that the intramuscular injection point was unusually high, and nearly all (17/20) acknowledged a similar perception when they were asked about this specifically (either compared with the previous dose administration or in relation to their theoretical expectation about the exact location of the point of injection). 

In three cases, infraclavicular lymphadenopathies with the same characteristics were also present. There were no signs of excess inflammation in the deltoid region. None of the cases had fever (> 38 °C). Splenomegaly was also absent. The rest of their physical examination was unremarkable. Cases are being followed up: all of them have improved clinically, and 15 completely resolved between 5 and 16 days since onset.

## Ethical statement

Informed consent from the patients was obtained before the clinical data collection. The patients agreed with the publication of the reported results.

## Discussion

Regional lymphadenopathies may constitute a sign of medical concern [2]. Although the most frequent cause is infection, neoplastic origin is also possible, particularly in certain locations such as the supraclavicular fossa [2]. Axillary lymphadenopathies after vaccination have been described for live attenuated vaccines such as *Bacillus* Calmette–Guérin [3], measles-mumps-rubella [4] or varicella [5] but also, more exceptionally, for inactivated vaccines. Axillary swelling or tenderness due to ipsilateral lymphadenopathy in the vaccination arm has recently been reported as an uncommon adverse reaction after the Pfizer–BioNTech vaccine (affecting up to one in 100 people) [6] and as very common after the Moderna vaccine (up to one in 10 people) [7]. However, supraclavicular lymphadenopathy had not previously been described as related to any of these vaccines.

An enlarged lymph node in the supraclavicular location is a concerning finding which may suggest either primary lymphatic malignancy, infection in the mediastinum or a metastatic malignancy from the abdomen, prompting complementary examinations [8,9]. Supraclavicular lymph nodes drain the neck but mainly they drain structures in the thorax and abdomen. A regional lymphadenopathy may develop in lymph nodes that drain the vaccination site. This phenomenon has been reported to appear in the first 2 weeks after vaccination and is related to local activation of the immune response. Less frequently, the location reported is supraclavicular, which has been described as associated with vaccination with influenza A(H1N1) [10] or human papillomavirus (HPV-9) vaccines [11].

Our cases developed supraclavicular adenopathy ipsilateral to the site of vaccination against COVID-19 without other signs or symptoms of interest. Although four cases had pre-existing conditions that might impact on their immune system, no specific relationship has been previously described between their underlying diseases and any specific adverse reactions to vaccination. Our hypothesis is that the immunisation technique may be related to the location of the adenopathy, with the supraclavicular territory and not the axillary being the most frequent drainage area in the event that the injection is administered at a higher location than recommended. The deltoid muscle is considered the optimal injection site for vaccines in the arm, concretely in the middle of the deltoid muscle, about two to three finger-widths below the acromion process. A range of injuries such as shoulder pain and dysfunction have been related to vaccination performed too high on the arm, where several anatomical structures exist, including the posterior circumflex humeral artery, the anterior branch of the axillary nerve or the subacromial–subdeltoid bursa [12]. All our patients where healthcare workers and most of them reported an unusually high point of puncture, not respecting the recommended distance between the acromion and the middle of the deltoid muscle. Nineteen of 20 cases were vaccinated in the left arm. This may simply be related to the fact that in right-handed people, vaccination is usually applied in the opposite arm, although it should be noted that left supraclavicular lymph nodes have more extensive drainage sites and drain more distant regions than nodes on the right side [8].

Our series may be just anecdotal. However, the recognition of supraclavicular lymphadenopathy as a self-limited inflammatory reaction in the context of ipsilateral arm vaccination may guide patients’ differential diagnosis and avoid unnecessary complementary examinations (blood analysis, locoregional echography, fine needle puncture aspiration, etc). Furthermore, if our hypothesis is true, the onset of this adverse reaction would be easily preventable with adequate training of vaccinators on the technique of administrating intramuscular vaccines. Taking into account the mass vaccinations taking place in Europe in spring, this information may be particularly relevant, not only to perform further surveillance of this event but also to prevent it through adequate vaccination technique and to consider this possibility in the differential diagnosis of an acute onset supraclavicular lymphadenopathy.

Our results cannot state specific incidence as these patients were not systematically referred to our centre and came from three different regions across Spain. The majority (90%) of mRNA vaccine doses distributed in Spain were Pfizer–BioNTech. We do not think that our findings have an impact on the safety profile of mRNA COVID-19 vaccines. Although the acute events following immunisation described in this series are time-related with vaccination, we cannot establish a causal relationship. However, our findings may call for further vigilance and the consideration of clinicians when evaluating acute supraclavicular lymphadenopathy after COVID-19 vaccination. According to our results, this constitutes a benign and self-limited condition related to injection-site technique.
